# A systematic approach to defining and verifying descriptors used in the Qualitative Behavioural Assessment of sows

**DOI:** 10.1017/awf.2024.6

**Published:** 2024-02-14

**Authors:** Sarah Ibach, Jen-Yun Chou, Monica Battini, Thomas D Parsons

**Affiliations:** 1 Swine Teaching and Research Center, University of Pennsylvania School of Veterinary Medicine, Kennett Square, PA, USA; 2 Pig Development Department, Animal & Grassland Research and Innovation Centre, Teagasc, Moorepark, Ireland; 3 Institute of Animal Welfare Science, University of Veterinary Medicine, Vienna, Austria; 4 Department of Agricultural and Environmental Sciences – Production, Landscape, Agroenergy. University of Milan, Via G. Celoria 2, 20133, Milan, Italy

**Keywords:** animal welfare, emotional state, on-farm welfare assessment, pigs, QBA, Welfare Quality ®

## Abstract

Qualitative Behaviour Assessment (QBA) is a welfare evaluation tool that uses a holistic approach to capturing an animal’s emotional state. Lists of QBA descriptors validated to assess pig welfare exist, but their definitions are often not described in peer-reviewed literature and the processes used to develop definitions are lacking. The objective of this study is to detail a systematic approach to creating clear definitions for a pre-existing fixed list of QBA descriptors and test their application. A fixed list of 20 descriptors from the EU Welfare Quality® assessment protocol for pigs was modified, and ten pig experts were recruited to assist with defining these descriptors in a focus group-style discussion. Half of the experts involved in creating descriptor definitions partook in a subsequent step, where the newly developed definitions were tested by implementing QBA on a video library of post-weaned sows selected to capture the breadth of sow behaviour. Experts displayed excellent agreement in identifying a PCA dimension interpreted as the valence of descriptors and good agreement for another reflecting arousal. Inter-observer reliability was also measured for each descriptor. Only two descriptors exhibited less than moderate agreement between experts whereas half of the descriptors evoked substantial agreement or better. These findings support our process to delineate clear definitions for a fixed list of QBA descriptors in pigs. This study is the first of its kind detailing the in-depth process of creating and verifying descriptor definitions for future use in sow welfare assessment.

## Introduction

Qualitative Behaviour Assessment (QBA) is a versatile animal welfare assessment tool that has been used in several farm species such as pigs (Morgan *et al.* 2014; Carreras *et al.* 2016; Schmitt *et al.* 2019), dairy cattle (de Boyer des Roches *et al.* 2018; Vindevoghel *et al.* 2019), beef cattle (Stockman *et al.* 2012), dairy buffalo (Napolitano *et al.* 2012), goats (Grosso *et al.* 2016; Battini *et al.* 2018) and sheep (Phythian *et al.* 2013; Willis *et al.* 2021). QBA facilitates capturing how individuals interact with their environment by recording “how the animal is behaving” instead of “what the animal is doing” (Wemelsfelder *et al.* 2000). It employs a holistic approach and enables an evaluation of animals’ presumed emotional state (Wemelsfelder *et al.* 2001). Today, good welfare is recognised as being more complex than the mere absence of negative experiences (Boissy *et al.* 2007) thus increasing the need to develop techniques used to identify presumed positive emotional states in animals. QBA’s value lies particularly in the technique’s ability to identify positive emotional states.

Welfare is assessed with QBA by scoring observed animals using various descriptors; descriptive terms such as ‘confident’ or ‘calm’ that detail an animal’s manner of interacting with its surroundings (Wemelsfelder *et al.* 2001). Two approaches can be employed in QBA studies: free choice profiling (FCP) and fixed list (FL) (Clarke *et al.* 2016). During FCP, assessors create their own list of QBA descriptors after observing an animal, while the FL approach provides assessors with a pre-determined list of descriptors. FLs of descriptors already exist for many farm animal species that have been developed, validated and published by experts (Welfare Quality® 2009; AWIN 2015). Currently, the most commonly used list of descriptors for pigs was published in the Welfare Quality® Assessment for Pigs (Welfare Quality® 2009). The Welfare Quality® project was a pan-European research project initiated in 2004. It aimed to develop standardised welfare assessment tools for farm animals that both supported animal management as well as offered benefits downstream in the value chain (Blokhuis *et al.* 2010). Their principles of good welfare include good feeding, housing, health, and appropriate behaviour (e.g. Welfare Quality® 2009). In the Welfare Quality® protocols, there are a range of various measurements to assess each principle with QBA being the technique used to assess the appropriate behaviour of animals. However, within these protocols, there are only instructions on how to conduct QBA and a list of descriptors. The process from which the descriptors were generated and defined, and more importantly, the definitions themselves, were never published or made available to the public. A lack of definitions can create difficulties in the understanding, training, and actual practice of QBA using FL to assess welfare, as the descriptors may be misinterpreted or misunderstood. Some studies have touched on the process to generate FL of descriptors in different species, such as donkeys (Minero *et al.* 2016), goats (Grosso *et al.* 2016), horses (Minero *et al.* 2018) and shelter dogs (Stubsjøen *et al.* 2020). The processes used to develop descriptor definitions have never been detailed for a FL of QBA descriptors in pigs.

The intention of this paper is to describe a detailed, systematic procedure for generating reliable and meaningful definitions for a pre-existing FL of QBA descriptors in pigs. The procedure consisted of six steps, starting with the modification of a pre-existing FL, recruiting an expert panel, generating definitions for the descriptors, voting on agreement, and later testing and verifying the newly developed definitions using QBA with subsequent statistical analyses. This systematic approach aims to decrease the ambiguity in interpretation of descriptors, allow for efficient training of assessors, and increase the standardisation of QBA findings both within and between studies.

## Materials and methods

### Ethical statement

The expert panel was recruited and consented to allow the use of their intellectual contributions for research purposes in exchange for an agreed upon monetary compensation. All data presented hereafter are anonymised and no personal information is provided. Video recordings were taken during a preceding study conducted in accordance with University of Pennsylvania’s Institutional Animal Care and Use Committee protocol #804656.

### The systematic approach

We developed a six-step process for creating clear and concise definitions of our FL QBA descriptors: Modify, Recruit, Define, Vote, Test, and Verify. These steps were created to breakdown defining descriptors into an easy-to-follow, transparent process.

### Modify: Adaptation of fixed-list descriptors

A pre-existing fixed list of 20 descriptors was modified from the Welfare Quality® assessment protocol for pigs (Welfare Quality® 2009) (Table 1). This fixed list of descriptors was chosen to facilitate longitudinal comparison across subsequent studies implementing the same descriptors. However, the original Welfare Quality® assessment protocol descriptor ‘social’ was removed and replaced with ‘curious’ (Duijvesteijn *et al.* 2014) as the current study focused on assessing pigs in an isolated context where social behaviour could not be expressed.

**Table 1.** A final list of descriptors modified from the Welfare Quality® assessment protocol for pigs, including groupings used during the focus group discussion based on word similarity

Grouping	Descriptors
1	Active, Curious, Lively, Playful, Positively occupied
2	Agitated, Distressed, Irritable, Tense
3	Aimless, Bored, Frustrated, Indifferent, Listless
4	Calm, Relaxed
5	Content, Enjoying, Happy
6	Fearful

### Recruit: Gathering the experts

Ten pig experts (researchers, veterinarians, and farmers, including three co-authors of this manuscript) were recruited to assist with the defining of descriptors and participate in a focus group-style discussion in May 2022. Experts with experience in different sectors of swine care, medicine, and research were selected to ensure diversity of perspectives when creating definitions.

### Define: Preparations

Prior to the focus group, experts were provided with a 6-min video to familiarise them with the process of QBA. The video content consisted of an introduction on the history and development of QBA, what QBA is, practical applications of QBA, and details on the differences between FCP and FL. After viewing the introductory materials, experts were instructed to submit preliminary definitions for each QBA descriptor based solely on their prior experience working with pigs. Descriptor definitions were to begin with the phrase, “A *descriptor* sow is…” to increase continuity between definitions. Experts were not given any information about the sows within the experiment prior to the focus group to maximise the likelihood of developing definitions with broad applicability beyond the scope of current study. The ten experts submitted one definition for each of the 20 descriptors. Definitions were compiled by the research team using R (R Core Team 2021) to analyse responses for common trends in words and phrases. A representative definition for each descriptor was created based on these trends in responses. In cases where submitted definitions included outliers from representative definitions, the outliers were collected and presented alongside representative definitions during the focus group, ensuring all points of view were reflected during discussion. Outliers were recognised as either definitions with keywords and themes minimally represented in trends or submissions that expressed concerns about the descriptor rather than a definition.

Representative definitions were provided to the expert panel one day prior to the focus group to allow the expert panel time to familiarise themselves with the definitions and serve as a starting point to facilitate discussion during the focus group. Experts were instructed to have the provided list of representative definitions readily available during the focus group.

### Define: Creating the definitions with the expert panel

Discussion was moderated by a co-author with previous experience training others in QBA. The focus group began with a brief introduction to the research project, presenting the experts with the background of the research team, the goals of the study, and the timeline for the focus group. Then, experts were read each of the 20 representative definitions one-by-one with an accompanying slide displaying the descriptor and definition. Once presented with the definitions, experts participated in a mock QBA session, where they scored four randomly selected, 1 min 40 s videos of sows within a novel arena taken from an ongoing study (Chou & Parsons 2022). Experts were instructed to complete QBA based on the representative definitions to test their efficacy.

Experts began their discussion of descriptor definitions following the mock QBA session. Experts were once again presented the 20 representative definitions, this time in six groupings based on descriptor similarities (Table 1). These groupings were created only as an aid to facilitate discussion and served no purpose beyond the focus group. Outlier definitions, including submissions expressing concerns rather than definitions, were presented to the experts prior to discussion of that descriptor. Experts were instructed to freely discuss each descriptor led by the moderator and reach a final consensus definition. For descriptors that did not reach a final consensus due to time limitations, definitions were formulated by the research team based on main discussion points from the experts.

### Vote: Quantifying agreement

Two weeks after the focus group, definitions as agreed upon during the focus group or formulated by the research team based on discussion were distributed to the experts. Anonymous voting forms were distributed online via Google Forms to quantify agreement for each final consensus definition. Experts were instructed to indicate whether they agreed or disagreed with the consensus definition. If they did not agree, experts could provide feedback through a subsequent short answer question on what they did not agree with. Descriptors were modified based on feedback as needed and re-voted on until 80% agreement was reached for each descriptor.

### Test: Trying out the definitions

Five of the original ten experts (excluding the three co-authors) volunteered to commit additional time to participate in the second phase to verify descriptor definitions (hereafter referred to as ‘verifying experts’). Verifying expert identities were anonymised so researchers would be blinded to the results. An identification code was self-created by prompting verifying experts to answer three questions the same way at the beginning of each form: What is your favourite animal? What is your favourite colour? And what is your favourite food? These responses generated a unique code used to track the responses of individual assessors while keeping their identities anonymous.

Verifying experts were provided with 12 pre-selected videos and instructed to view each one and complete the QBA. Videos used for QBA scoring were chosen from a library of videos compiled from the same previously mentioned study and were selected to represent a wide range of behaviours exhibited by sows based on previous ethological coding. For each video, verifying experts scored the 20 descriptors listed in alphabetical order using a visual analogue scale (VAS) ranging from 0 (minimum expression of the descriptor) to 125 (maximum expression of the descriptor), administered online via Zoho Forms (Zoho Corporation 2022), and were instructed to complete assessments based on the developed descriptor definitions. Verifying experts dragged a slider along each VAS to a point they felt appropriately represented the level of each descriptor displayed. QBA results were collected for statistical analysis.

### Verify: Statistical analysis

QBA scores from all five verifying experts were combined and analysed using R (R Core Team 2021). Principal Component Analysis (PCA) was applied to reduce the dimensionality of the QBA scores using the PCA function in the FactoMineR package with a correlation matrix and no rotation. The first two principal components with Eigenvalues greater than 1 that contributed to most of the variation were selected. The appropriateness of employing PCA to our data set was verified in two ways. Barlett’s Test of Sphericity (cortest.bartlett function in the psych package) was implemented to suggest sufficient correlation between variables. Kaiser-Meyer-Olkin factor adequacy analysis (KMO function in the psych package) was used to test the sampling adequacy of the model. Main factors or principal components (PCs) identified by PCA as well as individual descriptor scores were tested for inter-observer reliability using Kendall Correlation Coefficient W (KendallW function in DescTools library). Kendall’s W values can vary from 0 (no agreement at all) to 1 (complete agreement), with values higher than 0.6 showing substantial agreement (Landis & Koch 1977). Two linear mixed models were performed using the lmer function in the lme4 package with video as a fixed effect and verifying expert as a random factor to test the impact of each video on PC1 and PC2 scores. A one-way ANOVA was performed to test significant effects of each video.

## Results

### ‘Define’ step: Qualitative description of generating the definitions

Experts deliberated and agreed upon definitions for 17 out of 20 descriptors over the 4-h long focus group. The process started with the review of initial representative definitions of the descriptors to identify the parts of the representative definitions that would be included in final definitions.

Many of the initial representative definitions contained another descriptor. For example, the most common word in the initial definition of ‘happy’ was ‘relaxed’ and created issues in differentiating descriptors. The panel focused upon maintaining separation between descriptor definitions by not including descriptors in other definitions. Discussion was heavily focused on differentiations between positively valenced descriptors, specifically the differentiation between descriptors in Group 4 (‘calm’ and ‘relaxed’) and Group 5 (‘content’, ‘happy’, and ‘enjoying’). Consideration was given to combining descriptors from each grouping into one but was ultimately decided against. Definitions for the descriptors, ‘content’, ‘happy’, and ‘enjoying’ were not agreed upon due to time limitations on discussion. ‘Happy’ generated a large amount of discussion due to difficulty articulating what happiness looks like in a sow. For these descriptors, consensus definitions were created by the research team based on notes taken during the discussion of each descriptor and presented after the expert panel for subsequent voting to establish a consensus.

Experts also discussed when physical actions (behaviours) were needed in a definition versus instances in which describing how an animal is interacting with its environment sufficed. For example, the final definition for ‘curious’ states a sow “is inquisitive and interested in her environment [and] may actively approach objects and situations of interest or be investigating all aspects of where she is.” This definition contains descriptions of both *how* the sow acts (“inquisitive and interested in her environment”) and *what* the sow is doing (“actively approaches objects and situations of interest”, “investigating all aspects of where she is”). Careful consideration was given to the wording of these types of definitions to ensure the listing of actions was not criteria for fitting into a descriptor. The final list of descriptor definitions is presented below (Table 2).

**Table 2.** Final consensus definitions as agreed upon by the expert panel

Descriptor	Definition
Active	An active sow is not resting or sleeping and can be performing any type of motor activity.
Agitated	An agitated sow is restless and erratic. She may display a lot of movement or is in a hurry to do an action or change actions.
Aimless	An aimless sow is despondent, apathetic, and passive. She may be casually moving around without engaging with any specific element in her surroundings.
Bored	A bored sow is dull, unstimulated, and uninterested.
Calm	A calm sow is quiet, placid and without strong emotions.
Content	A content sow is showing signs of overall satisfaction. She is peaceful and comfortable.
Curious	A curious sow is inquisitive and interested in her environment. She may actively approach objects and situations of interest or be investigating all aspects of where she is.
Distressed	A distressed sow is upset. She may panic, actively search for escape, avoid stressors, or withdraw entirely. She may be anxious or in pain.
Enjoying	An enjoying sow shows pleasure in what she is doing, interacting with resources in her environment and voluntarily remaining in that environment.
Fearful	A fearful sow is startled, afraid, or terrified of a situation and may freeze or actively try to escape or retreat.
Frustrated	A frustrated sow is feeling discouraged in response to repeated attempts to achieve or change something without results, being prevented from achieving something, or repeatedly failing a task.
Happy	A happy sow is joyful, cheerful, and untroubled.
Indifferent	An indifferent sow is without interest in her environment, and she may not pay attention or react to stimuli.
Irritable	An irritable sow is easily reactive in a negative way towards any stimulus. She is uneasy and may be quick to flinch or show signs of annoyance.
Listless	A listless sow is lacking in energy, vigour, and enthusiasm. She may have a lost or sad expression or exhibit lethargic movements.
Lively	A lively sow is energetic, full of vigor, observant of her surroundings and may be quick to move.
Playful	A playful sow is fun-loving and gleeful. She may run, hop, spin, or manipulate items.
Positively occupied	A positively occupied sow is engaged in doing something she enjoys or needs, immersed in her surroundings in a focused, directed, unharmful and constructive manner.
Relaxed	A relaxed sow is at ease with her surroundings.
Tense	A tense sow is alert and uncomfortable. Her expression may be a worried or wary grimace and her posture may be stiff.

### Quantitative analysis of descriptor definitions

The first two principal components together explained 57.89% of the variation between videos (35.80 and 22.09% for PC1 and PC2, respectively). Table 3 shows loadings of each descriptor on the first two PCs.

**Table 3.** PCA of the QBA descriptors

Descriptors	PC1	PC2
Active	0.134	**0.703**
Agitated	−0.592	**0.611**
Aimless	−0.488	0.335
Bored	−0.392	0.437
Calm	0.342	−0.108
Content	**0.674**	0.421
Curious	0.455	**0.616**
Distressed	−0.579	−0.032
Enjoying	**0.815**	0.431
Fearful	**−0.603**	0.521
Frustrated	**−0.683**	0.550
Happy	**0.771**	0.500
Indifferent	−0.129	−0.331
Irritable	−0.591	**0.617**
Listless	−0.431	0.455
Lively	0.578	**0.629**
Playful	**0.753**	0.410
Positively occupied	**0.744**	0.405
Relaxed	**0.789**	−0.106
Tense	**−0.754**	0.482

Loadings with positive or negative values higher than 0.6 are shown in bold.

**Figure 1.**
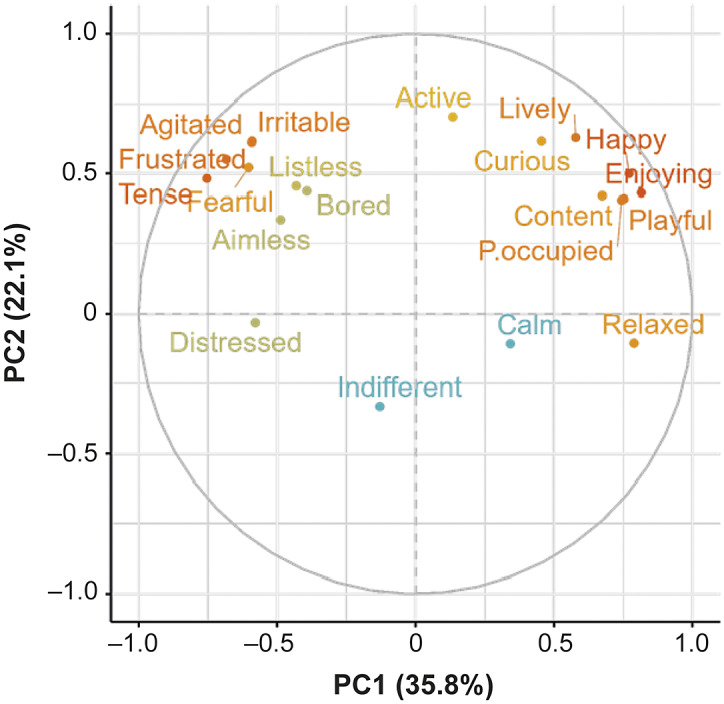
PC loadings for each descriptor. The colouring of the descriptors is representative of the strength of descriptors’ loadings, as determined by the factoextra package.

Many of the descriptors loaded strongly on the first PC and ranged from ‘enjoying/relaxed’ to ‘tense/frustrated’, suggesting that this component may describe the valence of sows’ affective states. The second PC seems more related to the level of arousal and ranges from ‘active/lively’ to ‘indifferent/calm’. The distribution of the descriptors across the first two PCs is shown in a loading plot (Figure 1). The valence and arousal of each descriptor are clearly defined along the axes, with negatively valenced descriptors near the left of the plot, positively valenced descriptors near the right, lower arousal descriptors near the bottom, and higher arousal descriptors near the top. The positioning of each descriptor on the loading plot is intuitive and lines up with the interpreted valence and arousal of each descriptor.

The appropriateness of employing PCA to our data set was verified in two ways. Barlett’s Test of Sphericity was highly significant (*c*^2^ = 1,106, df = 190; *P* < 0.0001) suggesting sufficient correlation between variables. Kaiser-Meyer-Olkin (KMO) factor adequacy analysis yielded an overall measure of sample adequacy of 0.8 on a scale of 0 (no sampling adequacy) to 1 (perfect sample adequacy). The lowest KMO score for an individual descriptor was 0.65 with 17 of the 20 scores being 0.7 or higher.

Agreement among the verifying experts ranged from almost perfect on PC1 (Kendall’s W = 0.91) to substantial agreement for PC2 (Kendall’s W = 0.66). High inter-observer reliability was also found between assessors for the individual QBA descriptor scores (Table 4) as only two out of the twenty descriptors exhibited less than moderate agreement (Kendall’s W < 0.4).

**Table 4.** Kendall’s W values for each of the QBA descriptors separately

Descriptor	Kendall’s W
Active	**0.68**
Agitated	**0.76**
Aimless	0.39
Bored	0.42
Calm	0.51
Content	**0.68**
Curious	0.49
Distressed	**0.68**
Enjoying	**0.81**
Fearful	0.39
Frustrated	0.59
Happy	**0.74**
Indifferent	0.48
Irritable	0.49
Listless	0.42
Lively	**0.86**
Playful	**0.88**
Positively occupied	**0.79**
Relaxed	0.55
Tense	**0.74**

Descriptors with Kendall’s W reflecting substantial agreement (W > 0.6) or better are shown in bold.

**Figure 2.**
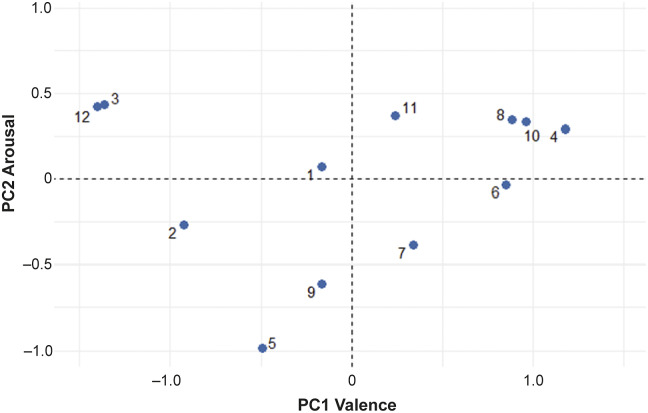
Mean PC values for each of the 12 selected videos. Videos are distributed along all four quadrants, indicating sows within each video were perceived as being in a different emotional state from one another.

Sows in the different videos were perceived by the observers as being in different emotional states. The PC dimension combinations are unique for each video and distributed across all four of the valence-by-arousal quadrants (Figure 2). Subsequent analysis with a linear mixed model revealed a significant effect of videos on both PC1 and PC2 (*P* < 0.001, respectfully) demonstrating the effectiveness of these definitions in differentiating emotional state in sows.

## Discussion

The objective of the present study was to document a transparent and understandable process for generating and then verifying reliable and meaningful definitions for QBA descriptors starting from a pre-existing fixed list of descriptors for pigs. Previous research into FL QBA has focused upon how the FL of descriptors were generated, which was typically carried out via a literature review and discussions among experts, but they did not provide specific details on how experts discussed the definitions and reached consensus (Grosso *et al.* 2016; Minero *et al.* 2018; Stubsjøen *et al.* 2020). This study is the first ever attempt to fully describe a systematic process for how a FL of QBA descriptors can be soundly defined. Further elucidating the process of defining the descriptors can prevent misunderstanding, improve the quality of assessor training and agreement, and strengthen the robustness of QBA as a measure of animal welfare.

QBA is a holistic approach that provides insight into the emotionality of an animal by assessing how an animal behaves, rather than simply what it is doing (Wemelsfelder *et al.* 2001; Andreasen *et al.* 2013). Given QBA’s holistic nature, the identification of commonalities in scoring patterns as identified through PCA is of more relevance than the use of individual QBA descriptors (Clarke *et al.* 2016). Our study shows an excellent agreement of verifying experts on PC1 that is more related to the valence of emotions, meaning that the experts consistently agreed on sows’ expression of negative and positive emotions. A good agreement was reached on PC2 that is more related to the arousal of emotions. The valence-arousal interpretation of emotional affect is frequently used in animal welfare and behaviour research (Mendl *et al.* 2010), and common across QBA studies in both pigs (Wemelsfelder & Millard 2009; Temple *et al.* 2013; Oldham *et al.* 2021) and other species (Diaz-Lundahl *et al.* 2019; Cooke *et al.* 2022; Travnik *et al.* 2022). In this study, the experts were able to distinguish whether the sows were in a positive or negative emotional state but were less likely to agree on arousal. A previous study investigating the implementation of FL descriptors in pigs, although without descriptor definitions, also reported better consensus for valence than arousal (Wemelsfelder & Millard 2009). However, our results showed higher inter-observer agreement compared to Wemelsfelder and Millard (2009) for both valence (W = 0.91 > W = 0.82), and arousal (W = 0.66 > W = 0.56).

We explored the agreement on each descriptor to better understand the implementation of our definitions. For half of the descriptors (ten of 20), the agreement of verifying experts was high and ranged from a low of W = 0.68 for ‘active’, ‘content’ and ‘distressed’ to a high of W = 0.88 for ‘playful’. Wemelsfelder and Millard (2009) also found high agreement in 22 out of 33 FL descriptors though it is important to note that only 16 of their 33 descriptors were included in our modified FL. Other studies implementing FL descriptors in other species, namely sheep and dairy cattle, found high agreement in six out of 13 descriptors (Muri & Stubsjøen 2017) and 15 out of 20 descriptors (Bokkers *et al.* 2012), respectively. Though good agreement was shown overall, our verifying experts showed lower agreement on certain individual descriptors. ‘Aimless’ is an example of this low agreement (W = 0.39). Difficulties in defining this descriptor already occurred during the focus group. Similar low agreement was found for ‘fearful’ (W = 0.39). Lower disagreement could also be due to the absence of certain emotional expressions that were not expressed by animals within the selected videos.

Pig experts were chosen for this study due to their familiarity with pigs and similar expertise, as previous studies investigating behaviour in shelter dogs found that welfare assessors with similar levels of experience working with a species assessed welfare in a similar manner (Munch *et al.* 2019). Since our newly developed definitions will be used to train a wide range of assessors in subsequent studies, we considered it coherent for the swine experts from the panel to complete the verification of these definitions. This also allowed for the possibility to fine tune the definitions, if necessary, before applying them to train other demographic groups to assess welfare, as the main task for our verifying experts was to test the definitions of the descriptors in QBA, rather than conduct a welfare assessment. However, it also is possible that this subset of observers who were also involved in the process of creating descriptor definitions might exhibit a more nuanced interpretation of the QBA descriptor definitions and, despite their similar backgrounds, yield some of the response variation we observed (*‘*aimless’, ‘fearful’, etc).

QBA is unique from traditional, ethological-based welfare assessments as it considers more than just an animal’s physical actions. During focus group discussion, experts spent sufficient time delineating physical actions a sow may be performing. Definitions containing physical actions were worded very carefully, particularly via inclusion of the word ‘may’ prior to any physical actions described. Concerns with the inclusion of physical actions arose when experts were worried that future QBA assessors using these descriptor definitions would believe a sow must be performing these actions to be considered as fitting into a certain descriptor, i.e. a curious sow must be actively approaching objects or situations of interest and investigating all aspects of where she is to be considered curious. We also acknowledge that as emotions are difficult to express in animals, especially when they can only be described using ‘human’ vocabularies resemblant of feelings (Mendl *et al.* 2022), diction plays an important role in the creation of descriptor definitions. It may appear that, in some instances, the definitions could lead to circularity when it comes to their interpretation (i.e. a ‘fearful sow is defined as being afraid, but it is also true that an afraid sow could be described as ‘fearful’). The circularity of the vocabulary surrounding emotional expression has been a long-standing discussion in semantics (Storm & Storm 1987), and it is argued that this debate can extend into discussion pertaining to animal emotion as well. Unlike defining an ethogram, where it is possible to use neutral terms to describe behaviours (Bateson & Martin 2021), it is not possible to define emotions using neutral terms. Therefore, definitions carefully deliberated upon by a group of pig experts using our systematic approach aim to serve as a guide to future assessors and help to ensure assessors would not think a sow must be performing an action to have a descriptor potentially apply to her during assessment.

Historically, animal welfare research has tended to focus upon studying negative affect (Yeates & Main 2008) since negative experiences are typically more profound and therefore easier to perceive and study than positive experiences (Boissy *et al.* 2007). However, QBA has been identified as being a promising technique for identifying positive affect (Temple *et al.* 2011; de Boyer des Roches *et al.* 2018; Schmitt *et al.* 2019) and is the only validated method for recording positive emotion in EU animal welfare assessment protocols (Welfare Quality® 2009; AWIN 2015). During the focus group, experts were able to agree upon all but three descriptor definitions: ‘content’, ‘happy’, and ‘enjoying’. Lack of consensus could be caused in part by the time limitations of the focus group and the similarity of the three descriptors, but also the difficulty in defining and recognising positively valenced emotions. Experts, indeed, spent most of their discussion focused on defining positively valenced descriptors. Although positive affect can be recognised with QBA, there remain challenges to expressing it in explicit terms, even by pig experts. Despite the potential difficulties, verifying experts displayed good agreement in the use of all three descriptors when implementing QBA (‘content’, W = 0.68; ‘happy’, W = 0.74; ‘enjoying’, W = 0.81), suggesting that definitions developed were sufficient. This also highlights the benefit of a systematic approach, as descriptors that may be harder to comprehend and easier to confuse were identified through the focus group discussion, which can be strengthened during the actual assessor training.

Two pairs of descriptors (‘agitated/irritable’ and ‘playful/positively occupied’) showed nearly identical PC loadings on PCs 1 and 2, causing almost complete overlap visually on the loading plot. This can be explained by the perceived similarity of the descriptors. During the expert panel, ‘agitated/irritable’ and ‘playful/positively occupied’ were identified by the research team and experts as being similar. During verification, ‘irritable’ displayed only moderate agreement between verifying experts (W = 0.49), while all other descriptors had favourable agreement (‘agitated’, W = 0.76; ‘playful’, W = 0.88; ‘positively occupied’, W = 0.79). While ‘agitated’ could be easily recognised in sows, it is possible this is not the case for ‘irritable’, a much more subtle emotion. Additionally, the barren environment of the novel arena may impact the sows’ ability to express the full repertoire of their behaviours and emotions, hence making certain emotions more difficult to assess (Haskell *et al.* 1996). We used videos of sows taken in a novel arena, in the absence of any cues revealing how they were housed, to avoid the possibility of observer bias as previously reported for behaviour observation and QBA (Tuyttens *et al.* 2014). Standardising the development of QBA definitions promises to be an essential first step toward a more detailed understanding of the possible role of observer bias in QBA studies. As we intended to use the WQ list without major alterations to enable subsequent cross-study comparison or meta-analysis, these four similar descriptors were not removed from the FL, though future studies could investigate the potential modification of these descriptors to further appropriate them based upon the assessed conditions or consider removing or adding descriptors.

### Animal welfare implications

QBA is an already well-studied and heavily used welfare assessment tool in many species. QBA’s value lies particularly in its ability to identify positive emotional states, as is often difficult to do with other methods of welfare evaluation. By refining and making clear the processes that go into the defining of FL descriptors in pigs, QBA as a welfare assessment tool will be better suited to assess positive aspects of pig welfare and increase the transparency and standardisation of the process.

## Conclusion

Our study set out to clearly define the procedures for generating reliable and meaningful definitions for a pre-existing set of fixed list QBA descriptors for sows. Our process detailed a systematic procedure used for creating and verifying descriptor definitions and is the first of its kind detailing this information in pigs. The results of this study promise a stronger, more reliable use of FL QBA for sow welfare assessment in the future.
